# Global response to pandemic flu: more research needed on a critical front

**DOI:** 10.1186/1478-4505-4-8

**Published:** 2006-10-13

**Authors:** Meng-Kin Lim

**Affiliations:** 1 Department of Community, Occupational & Family Medicine, Yong Loo Lin School of Medicine, National University of Singapore, Block MD3, 16 Medical Drive, Singapore 117597

## Abstract

If and when sustained human-to-human transmission of H5N1 becomes a reality, the world will no longer be dealing with sporadic avian flu borne along migratory flight paths of birds, but *aviation *flu – winged at subsonic speed along commercial air conduits to every corner of planet Earth. Given that air transportation is the one feature that most differentiates present day transmission scenarios from those in 1918, our present inability to prevent spread of influenza by international air travel, as reckoned by the World Health Organization, constitutes a major weakness in the current global preparedness plan against pandemic flu. Despite the lessons of SARS, it is surprising that aviation-related health policy options have not been more rigorously evaluated, or scientific research aimed at strengthening public health measures on the air transportation front, more energetically pursued.

## Background

Air transportation has undoubtedly been a boon to humankind – bringing together peoples, cultures and values, and profoundly changing the way we live. But it has also greatly aided the global transmission of infectious disease. In the old days, geographical distance provided a measure of protection as signs and symptoms had time to develop and those afflicted could be screened at border entry points. Today, with hardly an airport unreachable within 36 hours from any point on our planet, the speed – and pattern – of microbial movements has altered dramatically.

In 1992, the Institute of Medicine report *Emerging Infections: Microbial Threats to Health in the United States *correctly identified "microbial adaptation and change" and "expanding international travel and commerce" as two of the major factors contributing to disease emergence and re-emergence [1]. Severe Acute Respiratory Syndrome (SARS), in retrospect, epitomized this new model of disease outbreak. The previously unrecognized SARS-CoV coronavirus mysteriously surfaced in Guangdong Province, China, in November 2002, simmered there for three months, and arrived in Hong Kong on a jet plane. From that busy aviation hub, it quickly spread to Vietnam, Singapore, and Canada, eventually afflicting 27 countries and taking 813 lives [2].

## Learning from SARS

Thankfully, SARS did not progress to a full-blown pandemic as was widely feared. For reasons that are still unclear, the disease fizzled out, leaving us unsure as to whether we licked it or were just plain lucky. With avian flu now on everybody's mind, it is worth recalling the grim images of those dark days not so long ago – when some of the busiest airports in the world lay deserted as panic-stricken, would-be travelers stayed home. Anxious aircrew clamored for adequate protection at work [3] while medical and airline industry officials rejected the notion that the virus could be transmitted on airplanes – until the World Health Organization (WHO) weighed in to say that travelers seated within two rows of an infected person could be in danger. We now know that passengers sitting eight rows away are not any safer, and that out of a total of 40 commercial air flights investigated for carrying SARS infected passengers, five have been found to be associated with probable onboard transmission of SARS, involving 37 passengers in all [4].

The first in-flight transmission of SARS occurred in a female flight attendant who caught it from a family of three Singaporeans incubating the virus on a Singapore Airlines flight between New York and Frankfurt on 14 March 2003 [5]. Soon after, more cases were reported, such as when a cluster of thirteen passengers from Hong Kong was infected during an Air China Flight to Beijing on 15 March 2003, with a 72 year fellow passenger believed to be the source [6]. Then there was the pandemonium which broke when a certain 48-year-old man with symptoms of SARS was discovered to have flown on Lufthansa from Hong Kong to Munich, Barcelona, Frankfurt, London, Munich again, Frankfurt again, and back to Hong Kong before entering a hospital on his own accord. On 10 April 2003, the Hong Kong Department of Health had to desperately appeal for passengers and aircrew from all seven flights to consult their doctors [5].

As the fear of SARS became more contagious than the contagion itself, stock markets tumbled and billions of dollars were lost. Coming close at the heels of 9/11 and the Iraq war, SARS dashed hopes of recovery for the ailing airline industry. The latter is understandably not saying very much these days about any avian flu contingency plans they might have; one certainly hopes that appropriate preventive measures are being put into place. But that may be just the problem: What is the evidential base for effective public health interventions in the aviation industry, and how rigorously have the relevant aviation policy options been evaluated in the intervening years since the SARS episode [7]?

## Did we really learn?

Take thermal scanners for instance – first deployed in Singapore's Changi Airport and enthusiastically adopted by other "high-risk" airports around the world, in answer to the International Civil Aviation Organization's (ICAO) call for mass-screening of arriving and departing passengers and crews for raised temperature [8]. It was an innovative application of military technology to address an urgent need. To date, however, we are none the wiser regarding the sensitivity, specificity, or cost effectiveness of this screening tool for SARS, much less its usefulness for influenza. About all we know is that Canadian officials reportedly screened 1 million passengers with thermal scanners at an estimated cost of Can$7.55 million, without detecting a single case of SARS [9,10].

No one knows for sure what preventive measures all airlines and airports of the world should uniformly adopt in order to mitigate the spread of infectious diseases by air. The WHO's global influenza preparedness plan merely acknowledges, without elaboration, that "air travel might hasten the spread of a new virus, and decrease the time available for preparing interventions" [11] while ICAO's current website repeats the same general measures that it had posted for SARS [12]. With the threat of an influenza pandemic looming, which by all accounts will make SARS pale in comparison, all we have to go by today is the same generic advice on hand washing and personal hygiene for airline workers, and a negative assurance of sorts from the US Centers for Disease Control and Prevention (CDC) that "there is *no evidence *that avian influenza is spread through contact with baggage, packages, or other objects..." [13].

## Neglected front?

Apart from the desperate culling of affected poultry, much of the current global preparatory activities against avian flu pandemic revolve around surveillance, diagnostics, hospital infection control, vaccines production, and stockpile of antiviral agents. These efforts are necessary and laudable, but might they not also reflect the "medical" bias of existing paradigms? The SARS episode had highlighted the importance of enlisting travel industry workers and travelers as frontline fighters in the global response. If and when sustained human-to-human transmission of H5N1 becomes a reality, the world will no longer be dealing with sporadic avian flu borne along migratory flight paths of birds [14,15] but *aviation *flu – winged at subsonic speed along commercial air conduits to every corner of planet Earth. Surely any global battle plan against pandemic flu should entertain the notion of stopping the enemy at the gates, or along the corridors of its advance, before it reaches our homes, hospitals and clinics?

Alas, the 2005 WHO report *Avian influenza: assessing the pandemic *has dismally concluded that "If only a few countries are affected, travel-related measures, such as exit screening for persons departing from affected areas, might delay international spread somewhat, but cannot stop it. When large numbers of cases occur ... entry screening at airports and borders will have no impact" [16]. Granted, if a substantial portion of transmission occurs during the incubation or asymptomatic phase of disease, entry screening is unlikely to be effective in preventing or delaying an epidemic resulting from the importation of influenza [17]; and granted, the short time lag for scrambling upon discovery of a sentinel case will pose serious challenges to effective quarantine and contact tracing measures; but are we acquiescing on this critical front too readily?

## What must we do?

Sensible actions depend on knowing precisely what is going on, which in turn depends on good quality data. The fact of the matter is, we have simply not invested enough in the kind of multidisciplinary research needed, involving epidemiology [18], mathematical modeling [19], computational simulation [20], electronic tracking [21], and biological detection technology [22], to name a few, to elucidate the dynamics of microbial transmission associated with air travel, be it in aircraft cabins, toilets, or transit lounges. Four years after SARS, and we are no clearer regarding the complex spatial interactions of travelers converging on busy air terminals; or how best such human traffic may be channeled to minimize the risk of viral transmission; or what impact stringent screening impositions would have on passenger reaction and behavior. If the economic and wider arguments for maintaining continuity of air traffic flow (without which many nations could find their ability to keep going during a pandemic severely impaired) are not well researched and understood beforehand, arbitrary and capricious actions such as panic closure of borders, possibly leading to an abrupt global shut-down, could well result.

The current view is that under most scenarios, restrictions on air travel are likely to be of little value in delaying the proliferation of epidemics, unless almost all travel ceases very soon after epidemics are detected [23]. But if the technology for picking out passengers capable of transmitting deadly pathogens and setting off killer epidemics does not exist today, should we not be pursuing it as energetically as we do, the technology for stopping terrorists from boarding a plane? Against a conservatively estimated US$800 billion a year that a human pandemic of avian influenza could cost the global economy [24], not to mention the incalculable cost in terms of human lives [25], it seems incredible that the aviation lessons of SARS have not led to an acceleration of scientific research and health policy evaluation aimed at strengthening public health defenses on the air transportation front.

## Conclusion

To put things in perspective, we are engaged in a millennia-old, interspecies struggle between man and microbes. While the unseen enemy thrives because of its capacity for relentless adaptation and opportunistic spread, our own record of survival and progress owes much to the fact that at every critical turn, we have somehow managed to ask the right questions and looked hard enough at the right places for the right answers – be it in quarantine and vaccination strategies or an armamentarium of antibiotics and antiviral agents. In the coming epic battle against pandemic flu, the stakes have never been higher. If our strategies (read: health policies) are to work, they must be reliably informed by accurate intelligence (read: health research) which must cover all bases. Given that international air travel is the one feature that most differentiates present day transmission scenarios from those in 1918, it is surely relevant to ask, just how flu-ready are the airlines and airports of the world?

The call is for more scientific research devoted to this critical front. Two aspects deserve particular attention: (a) the science of transmission of infection between individuals and nations via air transportation and (b) the rigorous examination of policy options, based on the evidence and taking into consideration the economic trade-offs required. Resolving the tension between these aspects (and between the concerns of doomsday modelers and real-world policy makers in government, world health and air transport organizations) will improve the confusing impasse we seem to be in at present.

## Competing interests

The author(s) declare that they have no competing interests.

## References

[B1] Lederberg J, Shope RE, Oaks SC, Eds (1992). Emerging infections: microbial threats to Health in the United States. Committee on Emerging Microbial Threats to Health.

[B2] World Health Organization Cumulative Number of Reported Probable Cases of SARS from 1 Nov 2002 to 11 July 2003. http://www.who.int/csr/sars/country/2003_07_11/en/.

[B3] The Association of Flight Attendants Flight Attendants Demand Protection from SARS.

[B4] Mangili A, Gendreau MA Transmission of infectious diseases during commercial air travel. Lancet.

[B5] Lim MK, Koh D (2003). SARS and occupational health in the air. Occupational and Environmental Medicine.

[B6] Olsen SJ, Chang HL, Cheung TYY, Tang AFY, Fisk TL, Ooi SPL, Kuo HW, Jiang DDS, Chen KT, Lando J, Hsu KH, Chen TJ, Dowell SF (2003). Transmission of the Severe Acute Respiratory Syndrome on Aircraft. N Engl J Med.

[B7] Pickles H (2006). Using lessons from the past to plan for pandemic flu. BMJ.

[B8] Fitzsimons B ICAO takes action on SARS. Aviation International News. Paris. http://www.ainonline.com/Publications/paris/paris_03/pd2sarspg18.html.

[B9] St John RK, King A, de Jong D, Bodie-Collins M, Squires SG, Tam TW (2005). Border screening for SARS. Emerg Infect Dis.

[B10] Rothstein MA, Alcalde MG, Elster NR, Majunder MA, Palmer LI, Stone TH, Hoffman RE (2003). Quarantine And Isolation: Lessons Learned From Sars. A Report to the Centers for Disease Control and Prevention Institute for Bioethics, Health Policy and Law University of Louisville School of Medicine.

[B11] World Health Organization Department of Communicable Disease Surveillance and Response Global Influenza Programme (2005). WHO global influenza preparedness plan-The role of WHO and recommendations for national measures before and during pandemics. http://www.who.int/csr/resources/publications/influenza/WHO_CDS_CSR_GIP_2005_5.pdf.

[B12] Singh J, Finkelstein S (2005). Airport readiness for possible pandemic benefits from experience with SARS. ICAO Journal.

[B13] Centers for disease control and prevention Interim Guidance for Airline Cleaning Crew, Maintenance Crew, and Baggage/Package and Cargo Handlers for Airlines Returning from Areas Affected by Avian Influenza A (H5N1) January 13, 2006. http://www.cdc.gov/travel/other/avian_flu_airlines_cleaning_update_120505.htm.

[B14] Liu J, Xiao H, Lei F, Zhu Q, Qin K, Zhang XW, Zhang XL, Zhao D, Wang G, Feng Y, Ma J, Liu W, Wang J, Gao GF (2005). Highly pathogenic H5N1 influenza virus infection in migratory birds. Science.

[B15] Chen H, Smith GJD, Zhang SY, Qin K, Wang J, Li KS, Webster RG, Peiris JSM, Guan Y Avian flu: H5N1 virus outbreak in migratory waterfowl. Nature.

[B16] World Health Organization (2005). Avian influenza: assessing the pandemic threat. http://www.who.int/csr/disease/influenza/WHO_CDS_2005_29/en/.

[B17] Pitman RJ, Cooper BS, Trotter CL, Gay NJ, Edmunds WJ Entry screening for severe acute respiratory syndrome (SARS) or influenza: policy evaluation. BMJ.

[B18] Cox NJ, Subbarao K (2000). Global epidemiology of influenza: past and present. Annu Rev Med.

[B19] Rvachev LA, Longini IM (1985). A mathematical model for the global spread of Influenza. Mathematical Biosciences.

[B20] Grais RF, Ellis JH, Glass GE (2003). Assessing the impact of airline travel on the geographic spread of pandemic influenza. European Journal of Epidemiology.

[B21] UK Home office PublicTechnology.net. e-Borders will fence UK & use IT to track and identify passengers, Sep 30, 2004. http://www.publictechnology.net/modules.php?op=modload&name=News&file=article&sid=1831.

[B22] Scheller FW, Wollenberger U, Warsinke A, Lisdat F (2001). Research and development in biosensors. Current Opinion in Biotechnology.

[B23] Cooper BS, Pitman RJ, Edmunds WJ, Gay NJ (2006). Delaying the international spread of pandemic influenza. PLoS Med.

[B24] Brahmbhatt M (2005). Avian and human pandemic influenza–economic and social impacts.

[B25] CNN. Bird flu may kill 150 m, warns UN, 30 September 2005. http://www.cnn.com/2005/WORLD/asiapcf/09/29/birdflu.un/.

